# Simultaneous Determination of Different Polyamines and their Mono-Acetylated Derivatives in Gastric Tissue by HPLC with Post-Column Derivatization

**DOI:** 10.3797/scipharm.1001-02

**Published:** 2010-05-05

**Authors:** Muhammad Raza, Othman A. Al-Shabanah

**Affiliations:** 1 Department of Pharmacology and therapeutics, College of medicine, Qassim University, P.O.Box 6655, Mulaida 51452, Qassim, Saudi Arabia; 2 Department of Pharmacology, College of Pharmacy, King Saud University, Riyadh, Saudi Arabia

**Keywords:** Endogenous polyamines, HPLC, Post-column derivatization, Rat, Gastric tissue

## Abstract

A simple and highly sensitive procedure is described enabling the simultaneous determination of biogenic polyamines (PAs) and their related monoacetyl derivatives in stomach tissue. The method is based on HPLC using octane sulfonate as an ion-pairing agent employed in acetate buffers at pH 4.5. The application is accompanied with fluorescence detection followed by post-column derivatization with *o*-phthaldialdehyde at room temperature (20±0.5°C). N^1^- and N^8^-acetylspermidines (ASPD) can be determined with this method in the same run without performing any special procedures or pre-purification in concentrations exceeding 8.5 pmoles. The variability in reproducibility of the day-today precision and duplicate determination, and simultaneous determination of standard mixture and biological samples were found < 2%. The mean (± s.e.mean) retention times (n=12) for putrescine (Put), N^1^-ASPD, N^8^-ASPD, spermidine (Spd) and spermine (Spm) are 8.97±0.025; 17.64±0.063; 18.99±0.133; 28.20±0.070 and 39.81±0.098 min, respectively. The method was applied to determine PAs and specifically N^1^- and N^8^-ASPD in glandular part of stomach tissue of fasting rats (STFR) without any interference with endogenous aminoacids, histamine, and other reactive moieties. PAs and both mono-ASPD have been successfully determined in the STFR and the values are as follows: Put 37.2±10.1; N^1^-ASPD 5.88±0.48; N^8^-ASPD 4.43±0.94; Spd 750.7±22.7 and Spm 618.2±37.4 nmole/g of wet tissue. Information on gastric tissue polyamines and their acetylated derivatives may be useful in understanding the mechanism of drugs or agents that play some part in gastric ulcer production or its repair mechanisms.

## Introduction

The polyamines spermidine, spermine, their precursor diamine putrescine and their acetylated derivatives are considered to be universally distributed in the living cells and have been postulated to play an important role in the control of cellular growth. Polyamines are implicated to play an important role in cell proliferation, ulcer healing [1, 2], acid secretion inhibition [3] and gastroprotection by epidermal growth factor [4] that has been demonstrated to stimulate gastric mucus synthesis [4–7]. A correlation of polyamine levels of patients with inflammatory bowel disease had shown an increase in concentration of spermidine, N^1^- acetylspermidine and N^8^-acetylspermidine with increased inflammation [8] in the colonic epithelial cells and a lack of anti-inflammatory polyamine, spermine in severe ulcerative colitis. Another study [9] demonstrated that N^8^-acetylspermidine protected cerebellar granule cells from low K^+^-induced apoptosis which is p53 independent compared to parent polyamine.

There is an increasing interest in determining the polyamine concentrations in tissues and body fluids on routine basis. The role of tissue polyamines as a marker to screen ulcer status or to monitor the efficacy of therapy needs revision of procedures. The separation and/or determination of polyamines in various biological samples has been accomplished by several methods such as thin layer chromatography (TLC) [10–12], gas chromatography [12–13], ion exchange chromatography [14–18], enzymology [19], and fluorimetry [20–22]. TLC or other methods employing aminoacid analyzers have several drawbacks to their general use. TLC technique usually require extensive sample pretreatment and labor and variability in tissue sample contents and their excretory products can cause problem with the analysis.

Most of the published procedures for analysis of these amines by HPLC have relied on separation of tosyl [23–24] or dimethylaminonephthalene-1-sulphonyl derivatives [25–29]. However, these methods need either lengthy procedures or in complex gradient profiles it is desirable to carryout derivative purification prior to chromatography. Thus there is need for a specific assay of polyamines and their derivatives that separate them from interfering substances as well as structurally related compounds. It is well known that HPLC coupled with fluorescence detection is a powerful tool for the assay of polyamines. We report a specific modified HPLC-fluorescence assay method of Seiler and Knödgen [25] with modifications by Marchant *et al.* [30] for diamines, polyamines and their acetylated derivatives in pmole range in gastric tissue in a single run. This method involves post-column derivatization of the polyamines, their precursor diamine and acetylated derivatives, following HPLC separation on a reversed phase column that eliminates interference by some aminoacids, histamine and other compounds in the biological samples.

## Results and Discussion

**Fig. 1.**
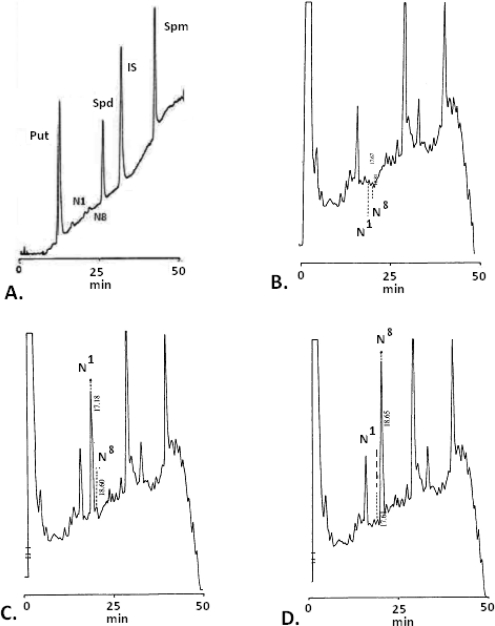
HPLC chromatograms showing putrescine, N^1^-acetylspermidine, N^8^-acetylspermidine, spermidine and spermine in gastric tissue extract of rat. A) A chromatogram showing standards and Internal standard (IS) B) Normal fasting rat gastric tissue extract. C) Normal fasting rat gastric tissue extract where N^1^-ASPD is spotted externally. D) Normal fasting rat gastric tissue extract where N^8^-ASPD is spotted externally.

Figures (1A–1D) show the separation of a mixture of polyamines and their related two monoacetylated polyamines directly from the tissue extract of the glandular part of the stomach. N^1^- and N^8^-acetylspermidines (ASPD) could be determined with this method in a same run without performing any special procedures or pre-purification in concentrations exceeding 8.5 pmoles. This method was also found to give a linear fluorescence response for increasing concentrations of other polyamines. The results show that the fluorescence quantum yield of the derivatives is different with different polyamines. The linearity of the fluorescence response was maintained even in the presence of other substances like aminoacids, histamine and other reactive species in the extracted biological samples. In order to minimize day-to-day variations in response it was found necessary to standardize the derivatization procedure as to time and temperature. Constructing a complete standard curve with each new stock solution minimized intra-assay variations in OPA reagent. The variability in reproducibility of the day-to-day precision and duplicate determination, and simultaneous determination of standard mixture and biological samples were found < 2%. The mean ±s.e. retention times (n=12) for Put, N^1^-ASPD, N^8^-ASPD, Spd and Spm are 8.97±0.025; 17.64±0.063; 18.99±0.133; 28.20±0.070 and 39.81±0.098 min, respectively (Table 1). The method has been applied to determine PAs and specifically N^1^- and N^8^-ASPD in glandular part of STFR without any interference with endogenous aminoacids, histamine, and other reactive moieties. PAs and both mono-ASPD have been successfully determined in the STFR and the values are as follows: Put 37.2±10.1; N^1^-ASPD 5.88±0.48; N^8^-ASPD 4.43±0.94; Spd 750.7±22.7 and Spm 618.2±37.4 nmole/g of wet tissue (Table 2).

**Fig. 2.**
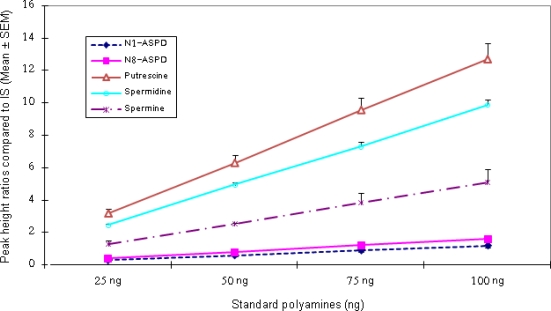
Calibration graph of relative peak height ratios of polyamines to an internal standard against their individual contents.

**Fig. 3.**
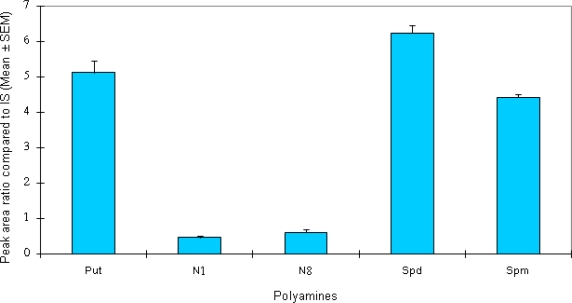
Relative peak area ratio of standard polyamines to internal standard (IS).

Figure 2 illustrates the calibration graphs obtained by the over-all procedure. Relative peak height ratios of Put, Spd, Spm, and N^1^-ASPD and N^8^-ASPD to an internal standard (1,7-diaminoheptane) in a standard mixture were plotted against the amount of each polyamine in the solution. For each polyamine, a good linear relationship was obtained in the concentration range shown in figure 2. Ten replicate determinations on an identical mixture containing Put (40.25 ng), Spd (63.62 ng), Spm (87 ng) and N^1^-ASPD and N^8^-ASPD (39 ng) gave standard deviation of 9.27, 13.02, 7.08, 16.99, and 10.79% respectively (Fig. 3).

The high-speed liquid chromatographic determination of Put, Spd, Spm, N^1^-ASPD and N^8^-ASPD by using post-column OPA deterivatization in STFR has been successfully performed. The utilization of this method has the following advantages:

1. The HPLC-fluorescence combination is highly sensitive and specific giving separation and detection of required polyamine contents.
2. The sensitivity of measurement is about 8.5 pmole for the rare acetylated polyamines in the gastric tissue samples, compared with other methods where these are not reported.
3. The method has the advantage of separating N^1^-ASPD and N^8^-ASPD as individual peaks from its precursor.

## Conclusions

Although discriminating between the common interfering substances in the samples, the method described here should prove valuable as a sensitive specific assay for polyamines and their acetylated derivatives that have been extracted from tissue samples or derived from biological fluids. Information on gastric tissue polyamines and their acetylated derivatives may be useful in understanding the mechanism of drugs or agents that play some part in gastric ulcer production or its repair mechanisms.

## Experimental

### Chemicals

Perchloric acid and *O*-phthaldialdehyde (OPA) were obtained from Fluka (Buchs, Switzerland), 1-octane sulfonic acid from Serva (Heidelberg, Germany), boric acid and sodium acetate trihydrate from Riedel de Häen (Seelze-Hannover, Germany), potassium hydroxide (p.a.) from Electro-Kemiska (Bohus, Sweden), 2-mercaptoethanol from E. Merck (Darmstadt, Germany), acetic acid, Brij-35 and methanol (HPLC grade) from BDH (Poole, England) and acetonitrile from Fisher Scientific Company (Newjersy, USA). The water was deionized, distilled and filtered through a 0.45μm Millipore filter.

### Animal Stocks

Experimental design and use of animals were approved by a local ethical committee (Research Ethics Committee, College of Pharmacy, King Saud University, Riyadh, Saudi Arabia). Male Wistar albino rats bred at Experimental Animal Care Center, King Saud University, Riyadh, all roughly 7–8 weeks old, weighing 150–180g, were used in this study. These animals were fasted for 48h in grid bottomed cages but allowed free access to water. The animals were killed by cervical dislocation and their stomachs were excised off the body, opened along the greater curvature, washed with saline and stored at −20°C until processed for HPLC use.

### Sample preparation

The glandular portion of the stomach tissue of fasting rats (STFR) was cut and placed in 4 volumes of ice-cold 0.25N perchloric acid containing 2.5μM 1,7-diaminoheptane (internal standard). Tissue was homogenized by using an Ultra-turrax^®^ (IKA-WERK, Janke & Kunkel GmbH & co. Staufen, Germany) homogenizer and centrifuged at 14x10^3^ rev. min^−1^ for 10 min at 4°C. The supernatant was decanted, filtered through a 0.45μm HA type filter (Millipore Corporation USA) and stored at 4°C till used for analysis.

### Analytical methods

The HPLC consisted of a multisolvent delivery system (Model, Waters 600E; Millipore corporation, Waters chromatography division, Milford, Massachusetts, USA), fitted with a liquid chromatograph injector (Waters, model U6K), a scanning fluorescence detector (model, Waters 470) and an integrator plotter (model, Waters 746, Data Module). A flow pump (Eldex Laboratories Inc. San Carlos USA) was used for external derivatization. The buffer A was 0.1 M sodium acetate adjusted to pH 4.5 with acetic acid and added with 10 mM octane sulfonic acid. The buffer B was 0.2M sodium acetate adjusted to pH 4.5 and containing 10 mM octane sulfonic acid (in its final concentration). Buffer B was mixed with acetonitrile in a 10:3 ratio prior to use. Both the buffers were purged with helium gas at 10 ml min^−1^. After elution post column derivatization was done by using OPA reagent prepared before use freshly by dissolving 44g KOH and 50g boric acid in distilled water, 3 ml (30% aqueous solution) of Brij-35 was added and made up to liter. To this solution 2 ml of mercaptoethanol and OPA 400 mg dissolved in 5 ml of methanol, was added in the said order. It was found advantageous to degas this working solution at −1 bar for 20 min before use. The flow rate for column elute and OPA reagent was adjusted to 1:1 ratio. A 25μl sample was injected and reversed phase HPLC was performed on a μ-Bondapak™ C_18_ analytical column (125Å, 10μm, 3.9x150 mm from Millipore Corporation, Milford, USA) at a flow rate of 1.5 ml min^−1^. and an ambient temperature of 20±0.5°C. The samples were eluted for polyamine separation with a linear gradient starting from 100% buffer A and decreasing the rate by 2% min^−1^ with a compensation from buffer B for the first 30 min whereafter this rate was changed to 4% min^−1^, till it reached to 100% buffer B and continued for another 5 min. A return to 100% buffer A was carried out at 45 min that stopped at 50 min. Post-derivatization, adducts were detected by fluorescence detector achieving excitation at 345 nm and measuring its emission at 455 nm.

### Evaluation of procedural performance

The accuracy of estimation procedure was evaluated by comparing the standard PAs obtained from commercial sources as indicated: 1,7 diaminoheptane (Fluka Chemika, Buchs, Switzerland); putrescine.2HCl, spermidine 3HCl, and spermine 4HCl (Sigma, St. Louis, M.O. USA); N^1^- and N^8^-acetylspermidine dihydrochloride (Winlab, Middlesex, UK). We have, so far, not come across any study that has assessed monoacetyl derivatives of spermidine (N^1^- and N^8^-acetylspermidine) simultaneously, while estimating other PAs in stomach tissue in experimental animals. Because of very low levels of acetylated derivatives the assessment was also performed by spotting biological samples with external supplements (Fig 1A–1D). The concentrations of PAs in the study samples were calculated from daily calibration curves. The variability in the calibration curve values was within the expected range (2.5–10% c.v.).

### Data analysis

Data are presented as the mean with the standard error (Mean±s.e.m)

## Figures and Tables

**Tab. 1.** Retention times of different polyamines in a mixture extracted from gastric tissue.

**Sample Polyamine’s mixture**	**Polyamine components**				
	**Putrescine**	**N^1^-acetylspermidine**	**N^8^-acetylspermidine**	**Spermidine**	**Spermine**
Retention time	8.97±0.025	17.64±0.063	18.99±0.133	28.20±0.070	39.81±0.098

Readings are mean (min) ± SEM of ten determinations.

**Tab. 2.** Polyamine contents in the rat gastric tissue.

**Treatment Dose**	**Putrescine nmol g^−1^**	**N^1^-acetylspermidine nmol g^−1^**	**N^8^-acetylspermidine nmol g^−1^**	**Spermidine nmol g^−1^**	**Spermine nmol g^−1^**
Control (Fasting)	37.2 ± 10.1	5.88 ± 0.48	4.43 ± 1.10	750.7 ± 22.7	618.2 ± 87.4

Readings are mean ± SEM of five determinations.
